# Moderately and Late Preterm Infants: Short- and Long-Term Outcomes From a Registry-Based Cohort

**DOI:** 10.3389/fneur.2021.628066

**Published:** 2021-02-12

**Authors:** Nikoletta Smyrni, Maria Koutsaki, Marianna Petra, Eirini Nikaina, Maria Gontika, Helen Strataki, Fotini Davora, Helen Bouza, George Damianos, Helen Skouteli, Sotiria Mastroyianni, Zoi Dalivigka, Argyris Dinopoulos, Margarita Tzaki, Antigone Papavasiliou

**Affiliations:** ^1^Second Department of Neurology, University General Hospital of Thessaloniki AHEPA, Thessaloniki, Greece; ^2^Third Pediatric Department, University General Hospital Attikon, Athens, Greece; ^3^Department of Orthopedics, Penteli Children's Hospital, Athens, Greece; ^4^Department of Pediatrics, Agia Sophia Children's Hospital, Athens, Greece; ^5^Department of Neurology, Evaggelismos General Hospital, Athens, Greece; ^6^First Department of Neurology, Aeginition Hospital, National and Kapodistrian University of Athens, Athens, Greece; ^7^Department of Neurology, Papageorgiou General Hospital, Thessaloniki, Greece; ^8^Department of Neonatal Intensive Care Unit, Agia Sophia Children's Hospital, Athens, Greece; ^9^Department of Neurology, Penteli Children's Hospital, Athens, Greece; ^10^Department of Pediatric Neurology, IASO General Hospital, Athens, Greece; ^11^Department of Neurology, Children's Hospital Aglaia Kyriakou, Athens, Greece; ^12^Rehabilitation Center, Children's Hospital Aglaia Kyriakou, Athens, Greece; ^13^Neonatal Intensive Care Unit, Elena Venizelou General Hospital, Athens, Greece

**Keywords:** cerebral palsy, moderately preterm, late preterm, premature birth, short term outcome, long term outcome, complication

## Abstract

**Background:** While most studies on the association of preterm birth and cerebral palsy (CP) have focused on very preterm infants, lately, attention has been paid to moderately preterm [32 to <34 weeks gestational age (GA)] and late preterm infants (34 to <37 weeks GA).

**Methods:** In order to report on the outcomes of a cohort of moderately and late preterm infants, derived from a population-based CP Registry, a comparative analysis of data on 95 moderately preterm infants and 96 late preterm infants out of 1,016 with CP, was performed.

**Results:** Moderately preterm neonates with CP were more likely to have a history of N-ICU admission (*p* = 0.001) and require respiratory support (*p* < 0.001) than late preterm neonates. Birth weight was significantly related to early neonatal outcome with children with lower birth weight being more likely to have a history of N-ICU admission [moderately preterm infants (*p* = 0.006)/late preterm infants (*p* < 0.001)], to require ventilator support [moderately preterm infants (*p* = 0.025)/late preterm infants (*p* = 0.014)] and not to have neonatal seizures [moderately preterm infants (*p* = 0.044)/late preterm infants (*p* = 0.263)]. In both subgroups, the majority of children had bilateral spastic CP with moderately preterm infants being more likely to have bilateral spastic CP and less likely to have ataxic CP as compared to late preterm infants (*p* = 0.006). The prevailing imaging findings were white matter lesions in both subgroups, with statistically significant difference between moderately preterm infants who required ventilator support and mainly presented with this type of lesion vs. those who did not and presented with gray matter lesions, maldevelopments or miscellaneous findings. Gross motor function was also assessed in both subgroups without significant difference. Among late preterm infants, those who needed N-ICU admission and ventilator support as neonates achieved worse fine motor outcomes than those who did not.

**Conclusions:** Low birth weight is associated with early neonatal problems in both moderately and late preterm infants with CP. The majority of children had bilateral spastic CP and white matter lesions in neuroimaging. GMFCS levels were comparable in both subgroups while BFMF was worse in late preterm infants with a history of N-ICU admission and ventilator support.

## Introduction

Prematurity is highly associated with neonatal mortality and morbidity and also constitutes a well-recognized risk factor for cerebral palsy (CP) and other lifelong effects on neurodevelopmental functioning and childhood/adulthood morbidity. According to the WHO, preterm birth is defined as any birth before 37 completed weeks of gestation and this can be further subdivided on the basis of gestational age (GA): very preterm (28 to <32 weeks), moderately preterm (32 to <34 weeks) and late preterm (34 to <37 weeks) (1, 2). So far, most studies on preterm birth have focused on very preterm infants as the group of preterm infants with the greatest risk of morbidity and mortality comprises those born at <32 weeks of gestation. However, this fact created a serious gap in the knowledge on the outcome of moderately (32 to <34 weeks GA) and late preterm infants (34 to <37 weeks GA) who represent the greatest number of infants born preterm. Over the past years there is a dramatic increase in studies focusing on moderately (32 to <34 weeks GA) and late preterm infants (34 to <37 weeks GA) highlighting that preterm infants born between 32 to <37 weeks of gestation are at higher risk of mortality and impaired short-term and long-term outcomes compared with infants born at term (3–6).

Several studies have shown that moderately and late preterm infants may suffer from respiratory distress syndrome (RDS) and other respiratory morbidities requiring respiratory support and neonatal intensive care unit (N-ICU) admission (7–9). Although respiratory issues tend to be transient in most moderately and late term neonates, some neonates may develop persistent pulmonary hypertension of the neonate (PPHN) or severe hypoxic respiratory failure (10). Additionally, moderately and late preterm infants are in higher risk of respiratory diseases and hospitalizations for respiratory infections in infancy and early childhood compared to their term peers (7, 11). Given that the last few weeks of gestation prepare the fetus for the transition from the intra-uterine life, the causes of respiratory distress observed in neonates even born after 32 weeks GA originate from the inability to adapt to the extra-uterine environment due to immature lung structure (7, 10). Neonatal seizures constitute another common complication in the newborn period. The incidence of neonatal seizures is typically associated with underlying brain injury and therefore adverse neurodevelopmental sequelae as well as high mortality rates with different aetiological profile in preterm compared to term infants and worse prognosis associated with younger gestational age (12, 13).

As far as long term sequelae is concerned, moderately and late preterm infants are reported to be at greater risk of CP and thus impaired motor function compared to term-born infants. The prevalence of CP rises with decreasing gestational age at birth and, additionally to motor manifestations, is frequently accompanied by cognitive and sensory impairment and epilepsy. The predominant abnormality among premature infants is spastic CP, mostly bilateral spastic (14). In terms of functional severity, Gross Motor Function Classification System (GMFCS) has been developed to provide a standardized classification of the patterns of motor disability in children with CP, with most of the patients distributed at GMFCS levels I-III compared to GMFCS levels IV-V (15–21). Several other assessments including the Bimanual Fine Motor Function (BFMF) Classification, the ABILHAND-Kids and the Manual Ability Classification System (MACS) have also been reported for fine motor function, but have not been studied extensively so far (22).

Abnormal neuroimaging findings are observed in the majority of children with CP. Based on the fact that CP is attributed to a non-progressive lesion or abnormality of the developing brain, the pathophysiology of CP depends on the time of occurrence of detrimental events during intra- and extra-uterine brain development. Disturbances during the first and second trimester of embryogenesis, when corticogenesis takes place, result in maldevelopments whereas disturbances during the third trimester when differentiation and “refining” events take place lead to predominant white matter injury mainly in the early third trimester and predominant gray matter injury in the late third semester when concerning cortical gray matter, basal ganglia and thalamus. However, given that CP is a clinical diagnosis, normal brain magnetic resonance imaging (MRI) does not exclude the diagnosis of CP (23–28). Previous studies confirmed that moderate and late preterm infants may exhibit important abnormalities such as severe intracerebral hemorrhage (ICH), ventriculomegaly and periventricular leukomalacia (PVL), albeit rarely, as well as smaller brain size, widespread white matter microstructural alterations and immature gyral folding compared with full-term peers (29–31).

Preterm birth has also been associated with an increased risk of epilepsy in childhood and intellectual disability with poorer neurodevelopmental outcomes of preterm infants at school age. The incidence of epilepsy has been shown to decrease with increasing gestational age but increases after 41 gestational weeks. In addition to intracranial hemorrhage and neonatal seizures, low Apgar score, N-ICU admission, respiratory support, antibiotic treatment during neonatal period and a major congenital anomaly have been associated with increased risk of epilepsy (32).

Concerning developmental problems, it has been shown that preterm children are at significantly increased risk for adverse neurodevelopmental outcomes and intellectual disability compared to termborn peers. However, despite the fact that both early preterm and moderately-late preterm children have greater rates of developmental problems compared to full-term born children before school entry, after school entry persistent developmental problems are observed solely in the subgroup of early preterm children, indicating that moderately-late preterm children may develop better adaptation capacities (33–35). Apart from gestational age, longitudinal growth in the first years of life has also been associated with neuropsychological functioning in moderately and late preterm children (36).

Given that moderately and late preterm infants represent a multitudinous, insufficiently studied and vulnerable to health and developmental problems population among preterm infants, we conducted a comparative analysis on the short- and long-term outcomes of moderately (32 to <34 weeks GA) and late preterm infants (34 to <37 weeks GA) born in Greece based on population-based data of the Greek CP Registry.

## Materials and Methods

In this retrospective comparative study, the participants' sample was drawn from the CP Registry of Attica-Greece. Data on 95 children born at 32 to <34 weeks GA and 96 children born at 34 to <37 weeks GA out of 1016 with CP, born between 1999–2006, were analyzed in terms of their need for N-ICU admission, respiratory support with endotracheal intubation or continuous positive airway pressure (CPAP) ventilation, and the occurrence of neonatal seizures during the first 72 h after birth. Moreover, the clinical classification of Cerebral Palsy (CP) in spastic CP (characterized by increased muscle tone and pathological reflexes, either unilaterally or bilaterally), dyskinetic CP (characterized by involuntary, uncontrolled, recurring, occasionally stereotyped movements of affected body parts) and ataxic CP (characterized by abnormal posture/movement and loss of muscular coordination) was assessed in children of both subgroups of moderately and late preterm infants (37). Furthermore, neuroimaging findings in MRI were categorized according to MRICS [MRI Classification System, proposed by the Surveillance of Cerebral Palsy in Europe (SCPE) network] (28). Additionally, gross motor function classification (GMFCS level) and bimanual fine motor function classification by assessing the child's ability to grasp, hold and manipulate objects in each hand separately (BFMF level), the incidence of epilepsy, intellectual disability [defined by an intelligence quotient (IQ) <70 in Wechsler Intelligence Scale for Children, fourth (WISC-IV) and fifth edition (WISC-V)] and hearing disability in childhood were studied as parameters of long-term outcome of moderately and late preterm infants (38–41).

The data were analyzed using the STATA 13 software package. Continuous variables (birth weight, Apgar score) were tested for normality using the Kolmogorov Smirnov test. Since they were not normally distributed, they were expressed as median values (range). Comparisons of continuous variables across groups of patients were based on Mann Whitney U test. When compared in more than two groups (e.g., MRI findings) Kruskall Wallis test was performed. Categorical data were presented as frequencies and percentages and were compared with chi-square or Fisher's exact test, as appropriate and *P*-values of <0.05 were a priori considered statistically significant.

Because of the fact that our study is retrospective, missing values were observed in several variables, due to incomplete data recording. All participants, though, had complete data in variables representing type of CP, GMFCS, and BFMF levels, the primary outcomes for the registry, and gestational age. Since our aim was to simply describe and compare the subgroups of patients with different gestational ages (moderately preterm and late preterm infants), no method for missing data was utilized.

## Results

### Moderately Preterm Infants

**Figure 1:**
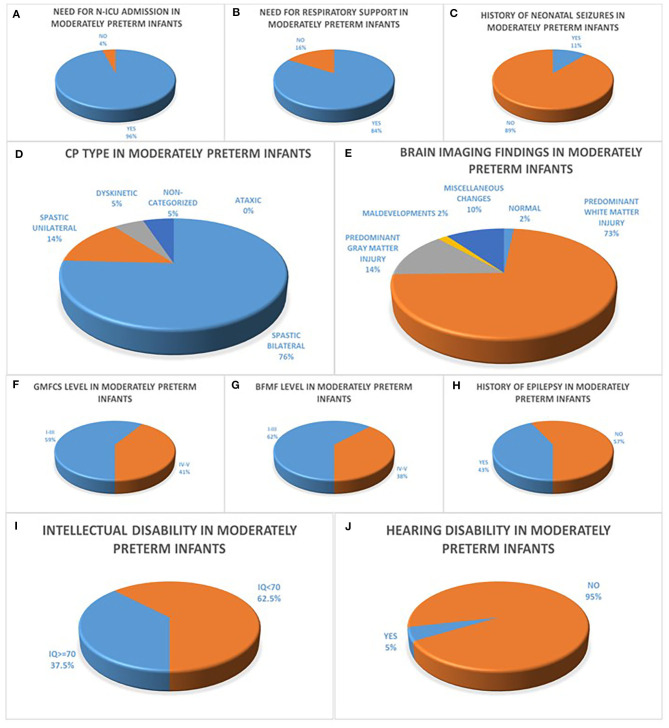
**(A–C)** refer to the neonatal history of moderately preterm infants born at 32 to <34 weeks GA with CP concerning the history of N-ICU admission **(A)**, the need for respiratory support **(B)** and the history of seizures during the first 72 h after birth **(C)**. CP characteristics of moderately preterm infants are depicted in diagrams **(D–J)** with the majority of infants with available data presenting with bilateral spastic CP **(D)**, predominant white matter injury in brain MRI **(E)**, GMFCS levels I–III **(F)**, BFMF levels I–III **(G)**, no history of epilepsy **(H)**, intellectual disability with IQ <70 **(I)** and no history of hearing disability **(J)**.

Of the 95 infants in the moderately preterm population, sixty-one percent (61%) were boys, mean Birth Weight (BW) was 1,780 g (870–3,400 g) and mean Apgar score was 9 (3–10). Within the population of moderately preterm infants, of the 94/95 infants with available data concerning the need for N-ICU admission ninety six percent (96%) had a history of N-ICU admission, of the 86/95 infants with available data concerning the need for respiratory support eighty four percent (84%) underwent endotracheal intubation or CPAP ventilation and of the 72/95 infants with available data concerning the history of neonatal seizures eleven percent (11%) presented with seizures during the first 72 h after birth. The predominant CP type among moderately preterm infants was bilateral spastic CP in seventy six percent (76%), followed by unilateral spastic CP in fourteen percent (14%) and dyskinetic CP in five percent (5%) while none of the patients (0%) presented with ataxic CP and five percent (5%) of the subgroup of moderately preterm infants were non-categorized. Among the 59/95 of moderately preterm infants with available brain MRI, the prevailing neuro-imaging findings were predominant white matter lesions in seventy three percent (73%), followed by predominant gray matter lesions in fourteen percent (14%), miscellaneous changes in ten percent (10%), maldevelopments in two percent (2%) and normal imaging findings in two percent (2%) of the patients. The motor function status of moderately preterm infants was assessed concerning gross motor skills with fifty nine percent (59%) classified as GMFCS Level I-III and forty one percent (41%) classified as GMFCS Level IV-V and fine motor skills with sixty two percent (62%) classified as BFMF level I-III and thirty eight percent (38%) classified as BFMF level IV–V. Furthermore, among the 84/95 of moderately preterm infants with available data, forty three percent (43%) had a history of epilepsy and fifty seven percent (57%) did not. Among the 80/95 of moderately preterm infants with available IQ score sixty two point five percent (62.5%) were classified as intellectually disabled (IQ <70) and among the 82/95 of moderately preterm infants with available hearing acuity test hearing disability was confirmed in five percent (5%) (Figure 1).

### Late Preterm Infants

**Figure 2:**
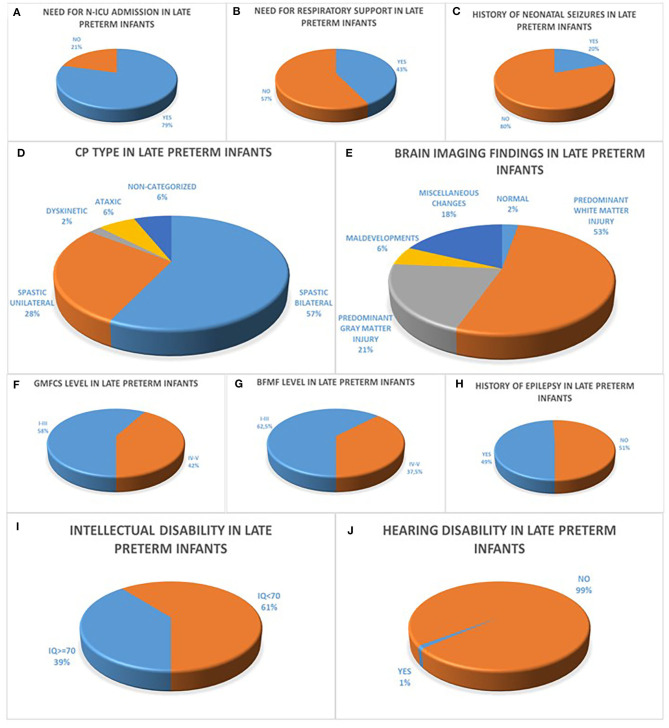
**(A–C)** refer to the neonatal history of late preterm infants born at 34 to <37 weeks GA with CP concerning the history of N-ICU admission **(A)**, the need for respiratory support **(B)** and the history of seizures during the first 72 h after birth **(C)**. CP characteristics of moderately preterm infants are depicted in diagrams **(D–J)** with the majority of infants with available data presenting with bilateral spastic CP **(D)**, predominant white matter injury in brain MRI **(E)**, GMFCS levels I–III **(F)**, BFMF levels I–III **(H)**, no history of epilepsy **(H)**, intellectual disability with IQ <70 **(I)** and no history of hearing disability **(J)**.

Sixty – four point six percent (64.6%) of all 96 late preterm infants were boys, mean BW was 2,310 g (850–3,800 g) and mean Apgar score was 9 (3–10). Within the population of late preterm infants, seventy nine percent (79%) were admitted in N-ICU during the neonatal period, of the 87/96 infants with available data concerning the need for respiratory support forty three percent (43%) of the patients required endotracheal intubation or CPAP ventilation and fifty seven percent (57%) did not and of the 81/96 infants with available data concerning the history of neonatal seizures twenty percent (20%) presented with seizures during the first 72 h after birth. The majority of late preterm infants had a diagnosis of spastic CP in eighty five percent (85%) classified as bilateral spastic CP in fifty seven percent (57%) and as unilateral spastic CP in twenty eight percent (28%) in the group of late preterm infants followed by ataxic CP in six percent (6%) and dyskinetic CP in two percent (2%) among late preterm infants while six percent (6%) of the patients were not categorized. Among the 72/96 of late preterm infants with available brain imaging with MRI, the prevailing finding was predominant white matter injury in fifty three percent (53%) of the patients, followed by predominant gray matter injury in twenty one percent (21%), miscellaneous changes in eighteen percent (18%), maldevelopments in six percent (6%) and normal imaging findings in two percent (2%) in the late preterm population. The motor function status of late preterm infants was assessed concerning gross motor skills with fifty eight percent (58%) classified as GMFCS Level I-III and forty two percent (42%) classified as GMFCS Level IV-V and fine motor skills with sixty two point five percent (62.5%) classified as BFMF level I-III and thirty seven point five percent (37.5%) classified as BFMF level IV-V, similarly to the subgroup of moderately preterm infants. In addition, among the 89/96 of late preterm infants with available data, forty nine percent (49%) had a history of epilepsy and fifty one percent (51%) did not. Among the 87/96 of late preterm infants with available IQ score sixty one percent (61%) were classified as intellectually disabled (IQ <70) and among the 91/96 of late preterm infants with available hearing acuity test hearing disability was confirmed in one percent (1%) (Figure 2).

### Birth Weight Is Related to Early Neonatal Outcome

**Figure 3:**
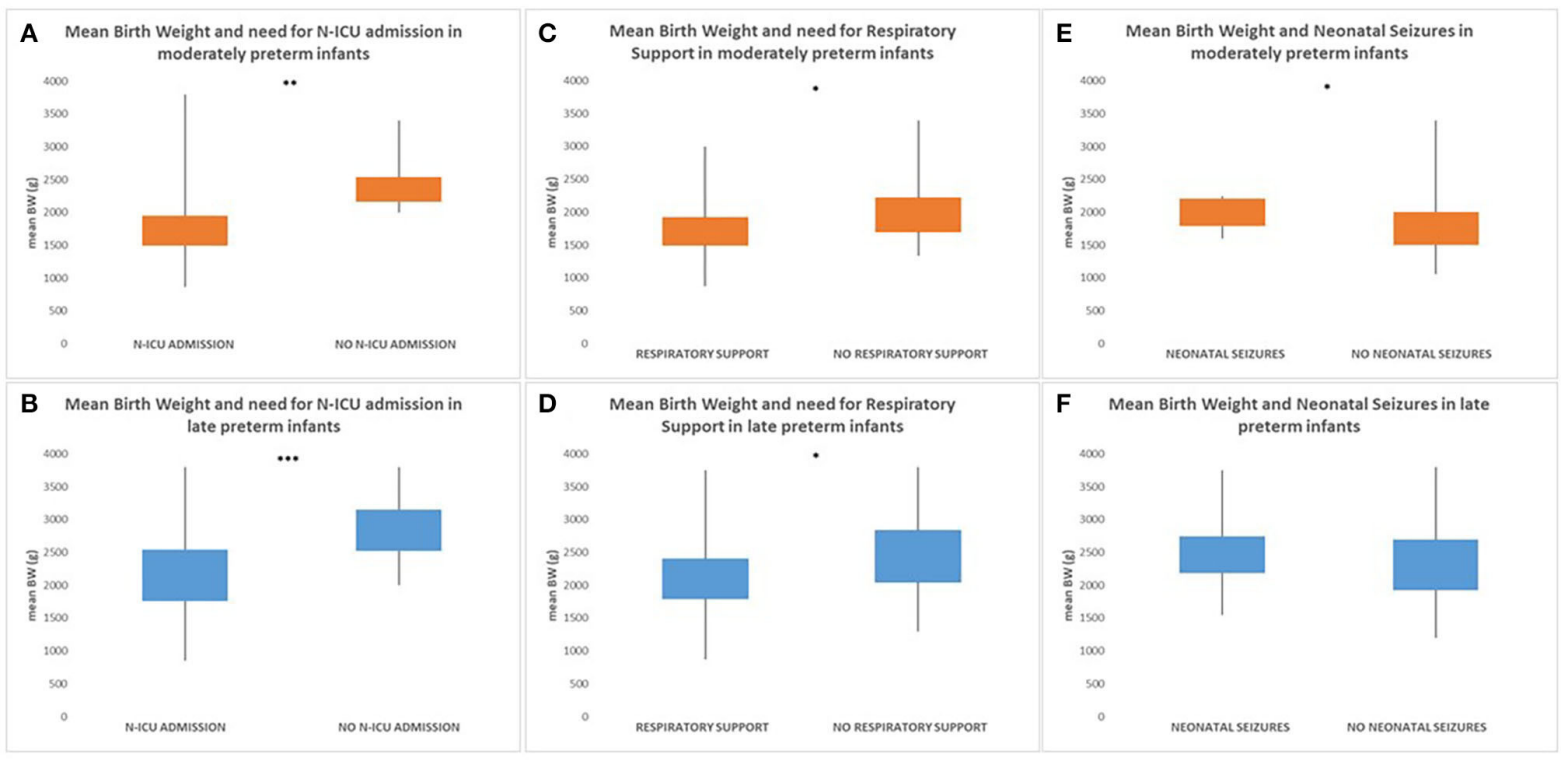
Both moderately and late preterm infants with lower birth weight were more likely to have a history of N-ICU admission [32 to <34 weeks GA (*p* = 0.006) **(A)**/34 to < 37 weeks GA (*p* < 0.001) **(B)**] and to require respiratory support [32 to <34 weeks GA (*p* = 0.025) **(C)**/34 to < 37weeks GA (*p* = 0.014) **(D)**]. Additionally, moderately preterm neonates with higher birth weight were more likely to have seizures during the first 72 h after birth with statistically significant difference compared to moderately preterm neonates with lower birth weight [32 to <34 weeks GA (*p* = 0.044)] **(E)**, whereas there was not statistically significant difference in the late preterm population [34 to <37 weeks GA (*p* = 0.263)] **(F)**. (*p ≤ 0.05, **p ≤ 0.01, ***p ≤ 0.001).

Birth weight was significantly related to early neonatal outcome with both moderately and late preterm infants with lower birth weight being more likely to have a history of N-ICU admission [32 to <34 weeks GA (*p* = 0.006)/34 to <37 weeks GA (*p* < 0.001)] and to require respiratory support [32 to <34 weeks GA (*p* = 0.025)/34 to <37 weeks GA (*p* = 0.014)]. Additionally, moderately preterm neonates with higher birth weight were more likely to have seizures during the first 72 h after birth with statistically significant difference compared to moderately preterm neonates with lower birth weight, whereas there was not statistically significant difference in the late preterm population [32 to <34 weeks GA (*p* = 0.044)/34 to <37 weeks GA (*p* = 0.263)]. In the moderately preterm population, the mean BW of neonates who required N-ICU admission was 1,770 g (870–3,800 g) whereas the mean BW of neonates who did not was 2,235 g (2,000–3,400 g), the mean BW of neonates who required respiratory support was 1,750 g (870–3,000 g) whereas the mean BW of neonates who did not was 2,025 g (1,340–3,400 g), the mean BW of neonates who had seizures during the first 72 h after birth was 2,075 g (1,600–2,250 g) whereas the mean BW of neonates who did not was 1,980 g (1,060–3,400 g). In the late preterm population, the mean BW of neonates who required N-ICU admission was 2,200 g (850–3,800 g) whereas the mean BW of neonates who did not was 2,750 g (2,000–3,800 g), the mean BW of neonates who required respiratory support was 2,150 g (870–3,750 g) whereas the mean BW of neonates who did not was 2,515 g (1,300–3,800 g), the mean BW of neonates who had seizures during the first 72 h after birth was 2,450 g (1,550–3,750 g) whereas the mean BW of neonates who did not was 2,300 g (1,200–3,800 g) (Figure 3).

### Gestational Age Is Related to Early and Late Outcome of Preterm Infants

**Figure 4:**
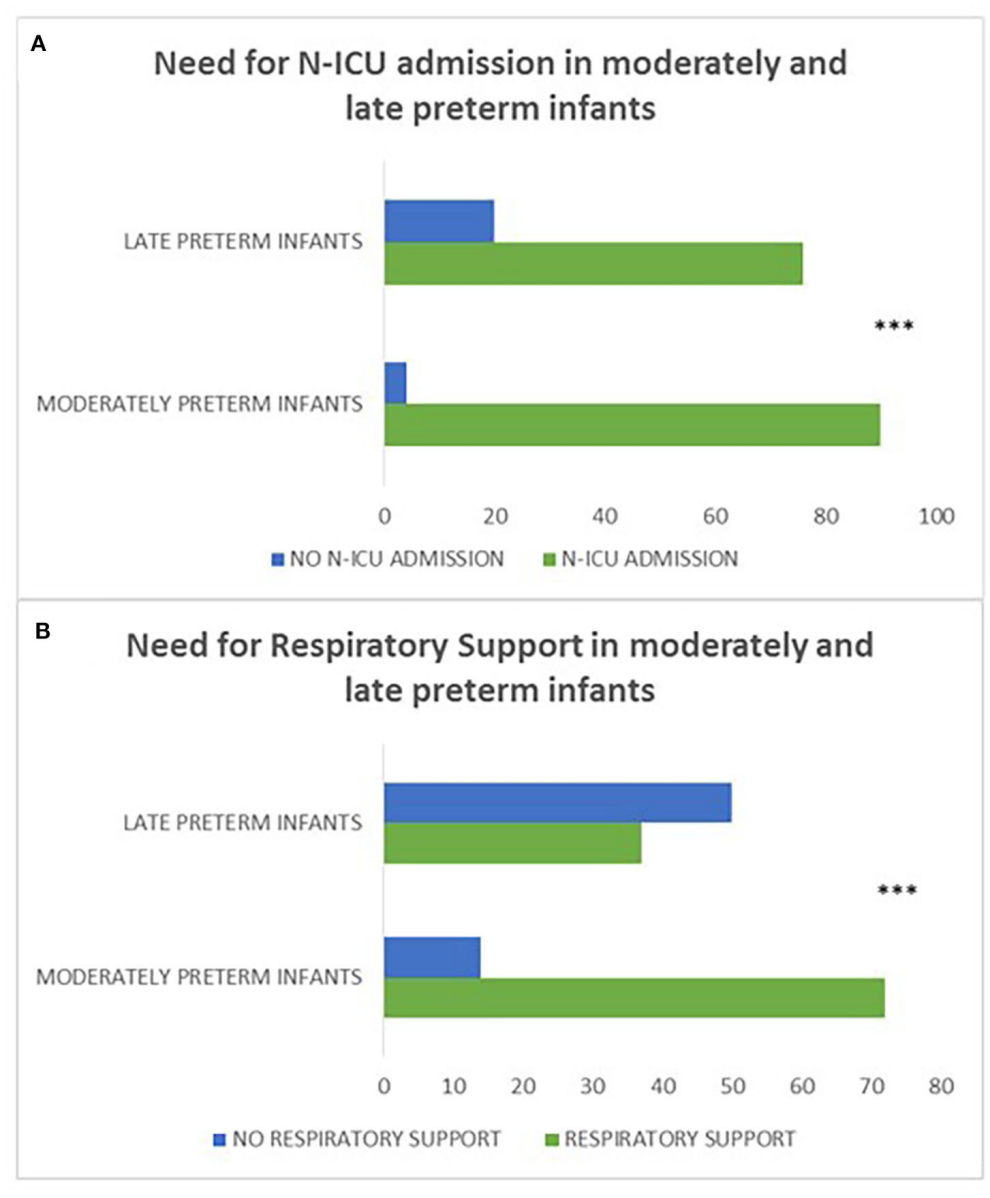
**(A, B)** refer to the history of N-ICU admission and need for respiratory support in moderately preterm compared to late preterm neonates. Moderately preterm neonates were more likely to have a history of N-ICU admission (*p* = 0.001) **(A)** and require respiratory support (*p* < 0.001) **(B)** compared to late preterm neonates. (***p ≤ 0.001).

Moderately preterm neonates were more likely to have a history of N-ICU admission (*p* = 0.001) and require respiratory support (*p* < 0.001) compared to late preterm neonates indicating that gestational age serves a crucial role in the neonatal outcome of preterm infants (Figure 4).

**Figure 5:**
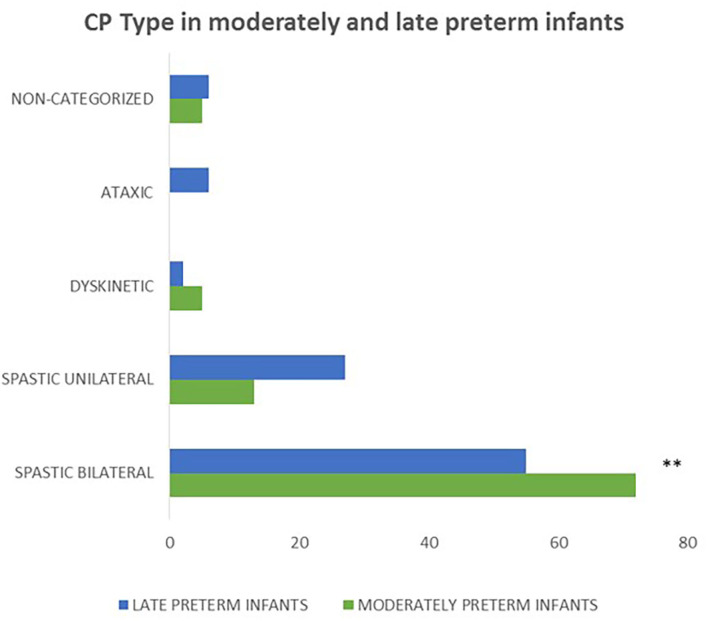
Concerning the correlation of gestational age and CP type, moderately preterm infants presented more frequently with bilateral spastic CP compared to late preterms (*p* = 0.006) and none of the moderately preterm infants had ataxic CP. (**p ≤ 0.01).

Concerning the clinical classification of CP, moderately preterm infants presented more frequently with bilateral spastic CP compared to late preterms (*p* = 0.006). Moreover, in the moderately preterm population none of the neonates had ataxic CP indicating that moderately preterm infants have less frequently ataxic CP compared to late preterm infants (Figure 5).

Among the 82/95 of moderately preterm infants and the 91/96 of late preterm infants with available data concerning auditory acuity, auditory disorder was observed in five percent (5%, 4/82) and one percent (1%, 1/91) respectively (*p* = 0.198), indicating that hearing disorder is not a major disability in infants of either subgroup.

**Figure 6:**
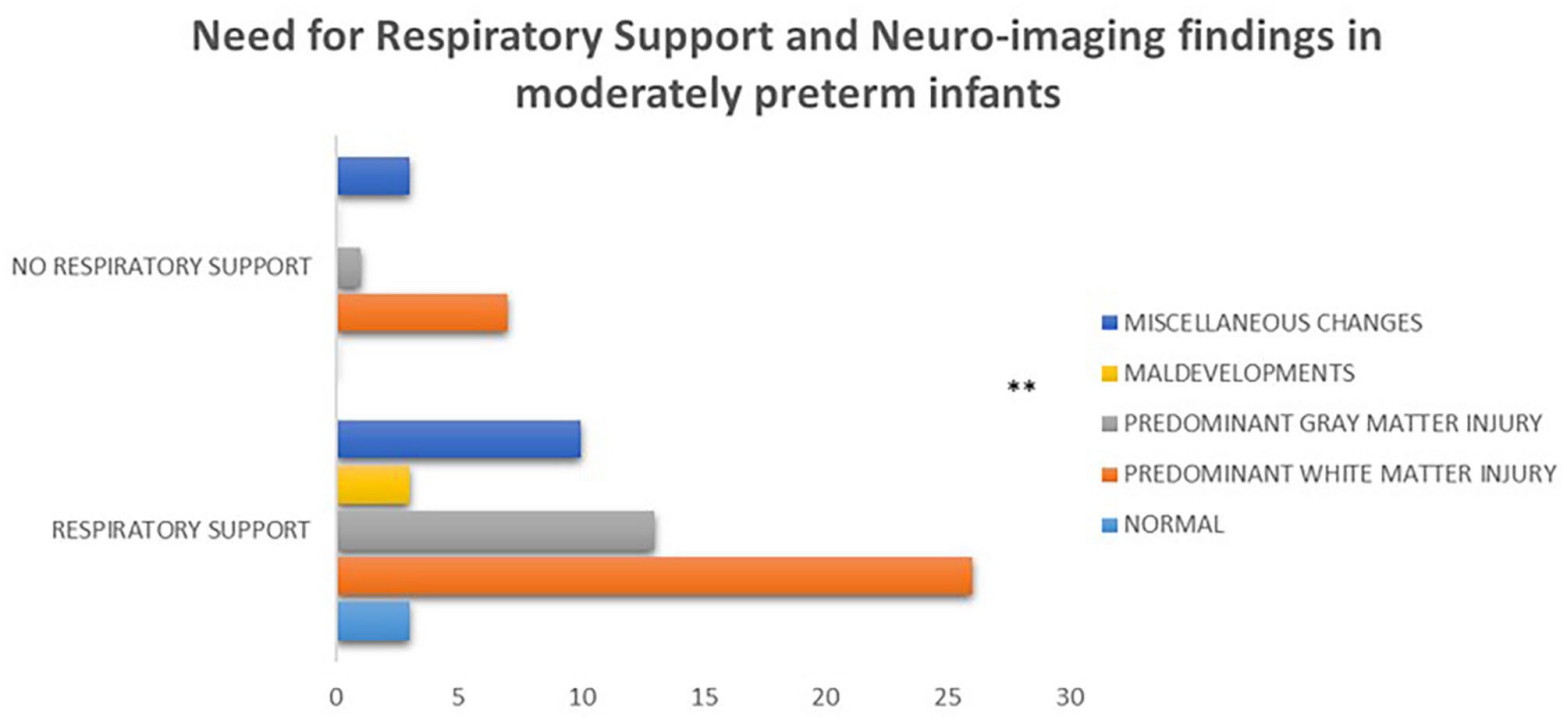
Among the 59/95 of moderately preterm infants with available brain imaging the prevailing findings were white matter lesions, with statistically significant difference between moderately preterm infants who required respiratory support and mainly presented with this type of lesion vs. those who did not and presented with gray matter lesions, maldevelopments or miscellaneous findings (*p* = 0.004). (**p ≤ 0.01).

Among the 59/95 of moderately preterm infants and the 72/96 of late preterm infants with available brain imaging the prevailing findings were white matter lesions in both subgroups (43/59, 73% and 38/72, 53% respectively), with statistically significant difference between moderately preterm infants born at 32 to <34 weeks who required ventilator support and mainly presented with this type of lesion vs. those who did not and presented with gray matter lesions, maldevelopments or miscellaneous findings (*p* = 0.004) (Figure 6).

**Figure 7:**
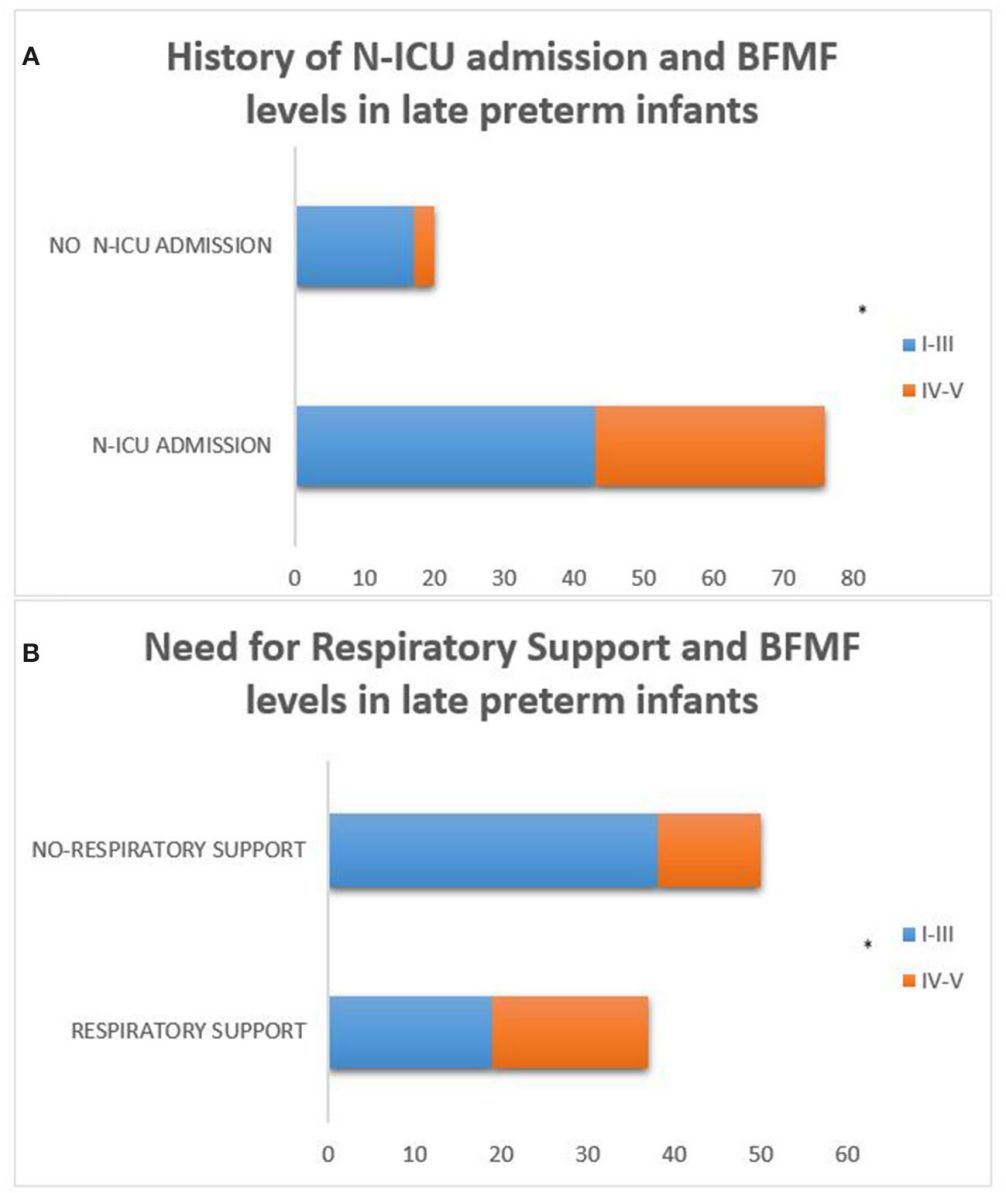
Late preterm infants who needed N-ICU admission and respiratory support as neonates were more likely to achieve less favorable fine motor outcomes according to the BFMF scale [*p* = 0.019 **(A)** and *p* = 0.017 **(B)**, respectively]. (*p ≤ 0.05).

In addition, within the population of late preterm infants, those who needed N-ICU admission and respiratory support as neonates were more likely to achieve less favorable fine motor outcomes according to the BFMF scale (*p* = 0.019 and *p* = 0.017, respectively) (Figure 7).

## Discussion

Birth weight turned out to be significantly related to early neonatal outcomes of both moderately preterm (32 to <34 weeks GA) and late preterm infants (34 to <37 weeks GA) as neonates of both subgroups with smaller birth weight were more likely to have a history of N-ICU admission, require respiratory support and not to develop seizures during the first 72 h after birth. The higher incidence of N-ICU admissions and need for respiratory support in moderately and late preterm neonates with lower birth weight in our study provides an indication of the predictive nature of birth weight and is similar to results of previous studies associating low birth weight with an increased risk of any emergency respiratory admission to hospital during the neonatal period, infancy and even later childhood until adulthood (11, 42–44).

In our cohort, moderately preterm infants were more likely to require respiratory support and have a history of N-ICU admission as neonates compared to their late preterm peers indicating that decreasing gestational age constitutes a negative prognostic factor for short term outcomes, in line with previous studies (7).

In both subgroups of moderately and late preterm infants, the predominant CP type was spastic CP (bilateral spastic CP followed by unilateral spastic CP), as expected based on current literature (14), and brain imaging revealed predominantly white matter lesions, as already highlighted in previous studies which documented intracranial hemorrhage (ICH), periventricular leukomalacia (PVL) as well as white matter microstructural alterations as the main neuroimaging finding in the population of moderately and late preterm infants (28–30).

Additionally, in the population of moderately and late preterm CP patients the classification of gross motor and fine motor function revealed comparable GMFCS and BFMF levels in both subgroups, with GMFCS and BFMF levels I-III in most patients. Several studies have been conducted so far concerning gross motor function in moderately and late preterm infants with CP indicating comparable results to ours, however, further studies are demanded in order to assess fine motor function in this population of infants as fine motor development of CP patients is poorly examined so far (16, 19–22).

Epilepsy and intellectual impairment were documented in high percentages, similar in both subpopulations of moderately and late preterm infants with CP, in accordance with current literature (32–35), whereas severe hearing disability was not observed in infants of either subgroup indicating further research needs as so far there are contrary existing literature data concerning hearing acuity in the population of moderately and late preterm infants (45, 46).

Our data support that moderately and late preterm infants constitute a population vulnerable for short-term complications and long-term unfavorable outcomes. Although, gestational age plays a crucial role for the short-term outcome and differentiates the clinical sequelae between moderately and late preterm infants during the neonatal period, the long-term outcomes of both subgroups of moderately and late preterm infants share common clinical features.

It is important to carry out further studies in order to follow up moderately and late preterm infants during prenatal neonatal period and childhood and to define prenatal and perinatal risk factors in order to improve obstetric and neonatal treatment practices that could lead to better short- and long-term outcomes.

Our study addresses the population of moderately and late preterm infants who represent the vast majority of infants born preterm and sheds light on short- and long-term outcomes of this subpopulation of CP patients providing data which appear to be consistent to current literature but also raise the necessity of further research.

## Data Availability Statement

The raw data supporting the conclusions of this article will be made available by the authors, without undue reservation.

## Ethics Statement

Ethical review and approval was not required for the study on human participants in accordance with the local legislation and institutional requirements. Written informed consent to participate in this study was provided by the participants' legal guardian/next of kin.

## Author Contributions

NS participated in the research design, the collection and statistical analysis of data and drafted the manuscript for intellectual content. MK, MG, HSt, FD, and GD participated in the collection of data. MP participated in the research design, the collection of data and revised the manuscript for intellectual content. EN participated in the research design, the collection and statistical analysis of data and revised the manuscript for intellectual content. HSk, HB, SM, ZD, AD, and MT participated in the diagnosis, treatment and follow up of patients, provided access to clinical data and revised the manuscript for intellectual content. AP conducted the research design, participated in the diagnosis, treatment and follow up of patients, provided access to clinical data, co-wrote the manuscript and revised the manuscript for intellectual content. All authors contributed to the article and approved the submitted version.

## Conflict of Interest

The authors declare that the research was conducted in the absence of any commercial or financial relationships that could be construed as a potential conflict of interest.
